# Modeling the enigma of complex disease etiology

**DOI:** 10.1186/s12967-023-03987-x

**Published:** 2023-02-25

**Authors:** Lynn M. Schriml, Richard Lichenstein, Katharine Bisordi, Cynthia Bearer, J. Allen Baron, Carol Greene

**Affiliations:** 1 University of Maryland School of Medicine, Institute for Genome Sciences, Baltimore, MD USA; 2 University of Maryland School of Medicine, Baltimore, MD USA; 3 Case Western Reserve University, Cleveland, OH USA

**Keywords:** Disease etiology, Diabetes, Asthma, Fetal alcohol syndrome, Pathophysiology, Environmental drivers, Genetics

## Abstract

**Background:**

Complex diseases often present as a diagnosis riddle, further complicated by the combination of multiple phenotypes and diseases as features of other diseases. With the aim of enhancing the determination of key etiological factors, we developed and tested a complex disease model that encompasses diverse factors that in combination result in complex diseases. This model was developed to address the challenges of classifying complex diseases given the evolving nature of understanding of disease and interaction and contributions of genetic, environmental, and social factors.

**Methods:**

Here we present a new approach for modeling complex diseases that integrates the multiple contributing genetic, epigenetic, environmental, host and social pathogenic effects causing disease. The model was developed to provide a guide for capturing diverse mechanisms of complex diseases. Assessment of disease drivers for asthma, diabetes and fetal alcohol syndrome tested the model.

**Results:**

We provide a detailed rationale for a model representing the classification of complex disease using three test conditions of asthma, diabetes and fetal alcohol syndrome. Model assessment resulted in the reassessment of the three complex disease classifications and identified driving factors, thus improving the model. The model is robust and flexible to capture new information as the understanding of complex disease improves.

**Conclusions:**

The Human Disease Ontology’s Complex Disease model offers a mechanism for defining more accurate disease classification as a tool for more precise clinical diagnosis. This broader representation of complex disease, therefore, has implications for clinicians and researchers who are tasked with creating evidence-based and consensus-based recommendations and for public health tracking of complex disease. The new model facilitates the comparison of etiological factors between complex, common and rare diseases and is available at the Human Disease Ontology website.

## Background

Figuring out causality of human diseases is akin to deciphering an enigma, wrapped in a mystery, composed of genetic and environmental riddles. Expanding our understanding of disease etiology based on the integration of multifactorial genetic, environmental, and lifestyle factors holds the potential for revealing biological pathways of disease, advancing our understanding of the polygenic basis of complex diseases, enhancing diagnostic capabilities, and enabling therapeutic development [1]. Understanding the full complexity of disease etiology is essential in order to provide the best management of patients and for researchers to develop novel therapies and preventive measures. Understanding complex disease variability across all populations (age, gender, and ethnicity) is essential in order to fully understand disease drivers, progression and variability in treatment efficacy and clinical outcomes. One goal of the Human Disease Ontology (DO) is to convey our understanding of disease etiology, at all levels of complexity, for clinicians and researchers, providing a conduit for representing and communicating our understanding with each other.

Representing the complex and variable biologic pathways of disease is necessary both for the clinician treating the individual patient, to understand the etiology of their disease in order to provide the best management, and for the researcher investigating the cause of disease, to develop new and improved therapies and inform preventative measures [2]. The imperative to advance our understanding has been heightened during the COVID-19 pandemic, as adults with complex diseases (such as obesity, cancer, chronic kidney disease, and chronic lung disease including COPD, asthma, interstitial lung disease, cystic fibrosis, and pulmonary hypertension) have an increased vulnerability to get severely ill from COVID-19 [3, 4].

The Human Disease Ontology represents 11,003 human diseases [5](June 2022 release) classified by etiology following disease community guidelines (e.g., cancers—WHO classifications; mental health diseases—DSM; genetic diseases—OMIM) [6, 7]. The DO codifies hierarchical relationships between diseases based on shared phenotypes, symptoms, cells of origin, anatomical location, mode of inheritance, structural variants, or transmission method for common and rare diseases. The DO actively integrates new clinical knowledge, refining and augmenting disease classifications to enhance our understanding of the complexities of human disease etiology and thereby enhance DO’s utility as a diagnostic tool for clinicians and researchers. The addition of genetic and cancer molecular subtypes provide novel access to our understanding of complex disease that should lead to insights into disease prevention and disease intervention.

Here, we present the development of an extended etiology model to capture the complexity and variable biological pathways of diseases in the Human Disease Ontology. This work was developed to address the challenges of classifying complex diseases in a way that recognizes the evolution and limitations of our current understanding. This model integrates current perspectives and facilitates the integration of evolving perspectives for chronic disease diagnosis and treatment. The aim of this work is to enhance users capabilities to examine and compare key etiological factors between diseases.

### Complex disease modeling

**Fig. 1:**
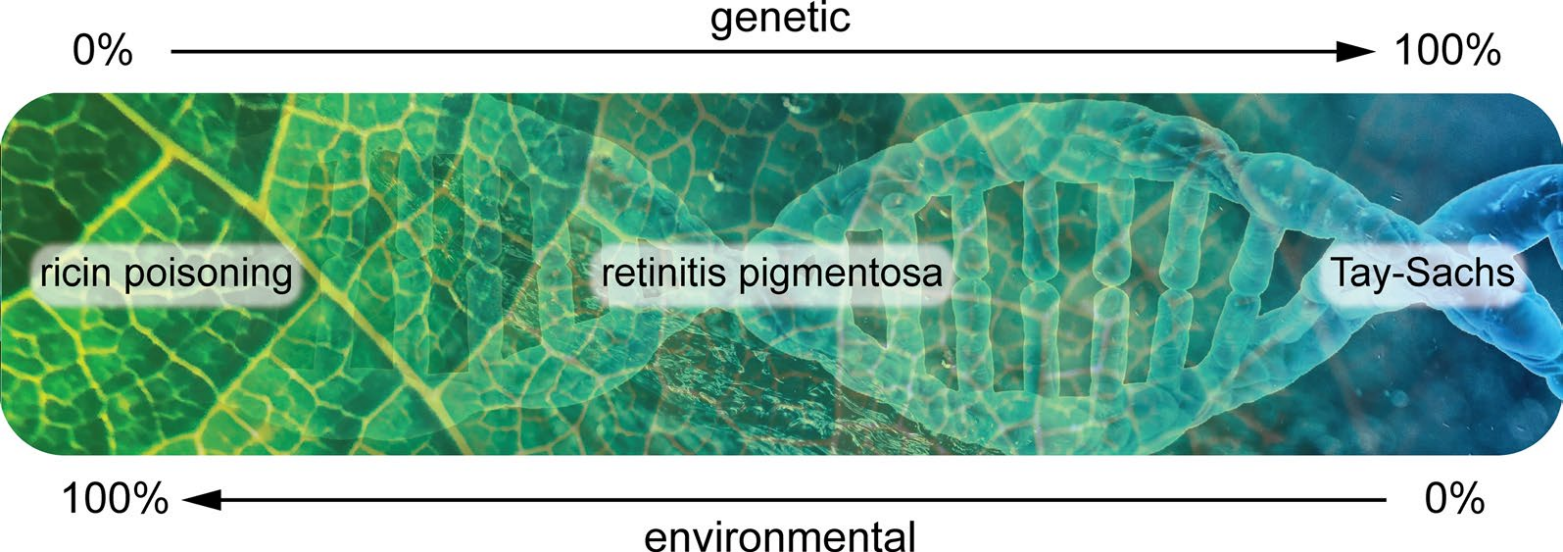
Spectrum of disease genetic etiology and environmental drivers. Examples of diseases that result from entirely environmental, a mixture of environmental and genetic (multifactorial), or entirely genetic etiology

Modeling the underlying mechanisms of complex disease [8, 9] requires a reassessment of approaches to address the complex interactions of genetic, genomic, environmental, and physiological mechanisms of complex disease. Modeling must recognize the environmental and genetic spectrum of complexity from single gene diseases with little or no modification by the environment (such as Tay Sachs and Huntington disease) to certain infections, poisons, and trauma that impact each human in similar fashion regardless of age or genetics (Fig. 1).

Therefore, modeling must appropriately represent disease etiology across the environmental-genetic spectrum (Table 1) from multigenic diseases (e.g. Prader-Willi) that do not have an environmental etiology driver, but may have environmental contributions that modify expression, to diseases involving a specific environmental driver (e.g. thalidomide embryopathy—resulting from in utero thalidomide exposure occurring between 20 and 36 days after fertilization) [10], and diseases resulting from a combination of environmental factors and certain genetic polymorphisms (e.g. Gilbert’s Syndrome and paracetamol or neonatal opiate withdrawal syndrome) to conditions (.e.g. ricin poisoning) resulting primarily from environmental drivers.

**Table 1:** Categories of genetic and environmental contributions in complex diseases. Genetic—Environment Spectrum

Disease	G/E Spectrum	Environmental Driver/Trigger	Genetics
Tay-Sachs [11]	G	None	Hexosaminidase A gene
Iminoglycinuria [12]	G	None	Solute carrier family genes
AMED syndrome [13]	G	None	Alcohol dehydrogenase genes
Prader-Willi (deletion) [14]	G	None	Deletion of genes on Chromosome 15
Prader-Willi (uniparental disomy) [15]	G/E	Intracytoplasmic sperm injection (ICSI)	Maternal uniparental disomy of Chromosome 15
alpha-1 antitrypsin [16]	G/E	Tobacco smoke, chemicals and dust impact severity	*SERPINA1* (serine protease inhibitor) gene
spina bifida [17]	G/E	Folate deficiency	Methylenetetrahydrofolate reductase (MTHFR) gene
retinitis pigmentosa [18]	G/E	UV light exposure	> 60 genes identified
myopia [19]	G/E	Near work, outdoor exposure	> 27 genes identified
fetal alcohol syndrome [20]	G/E	Alcohol	None, maternal ability to metabolize alcohol
ricin poisoning [21]	E	Ricin	None

*G* genetic, *G/E* genetic and environmental, *E* environmental

## Methods

### New approach to model complex diseases

**Fig. 2:**
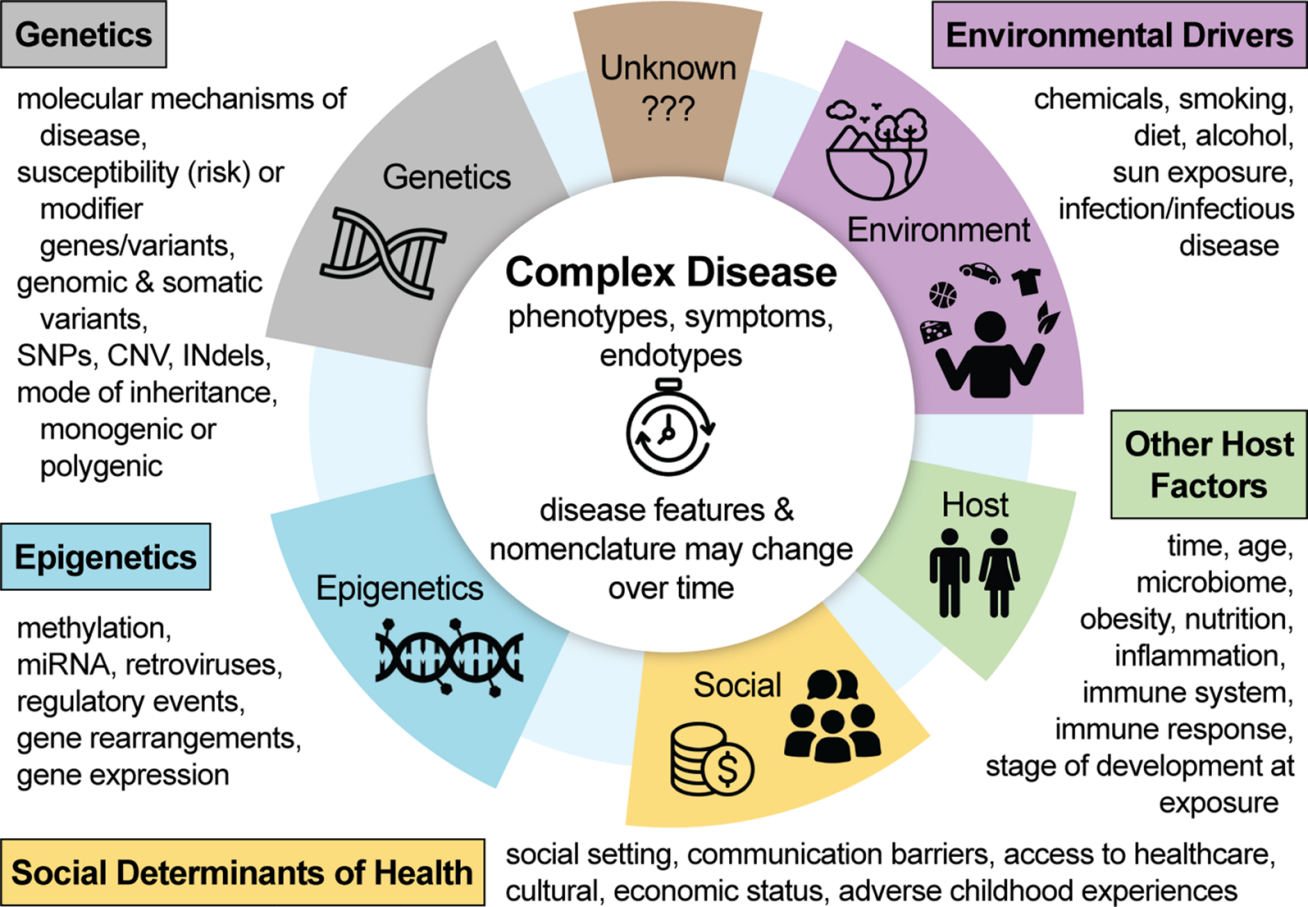
Modeling the complexity of disease etiology. Encompassing genetics, epigenetics, social determinants of health, environmental drivers and other host factors

While genetic susceptibility factors to complex diseases such as asthma, osteoarthritis, Parkinson’s, epilepsy, migraines, systemic lupus erythematosus, and cancers provide mechanistic insights, occurrence of the disease often depends on the proverbial second ‘environmental driver’ shoe to drop. Unraveling this complexity necessitates expanding our understanding of the interactions of genetic and environmental risk factors and the extent of their contributions towards disease. In order to devise new tailored therapies for precision medicine, we need a flexible system to represent our understanding of the pathophysiology of complex disease which includes the interaction of genes with infectious diseases and environmental challenges. Recognizing that one model does not fit all, the next step is to recognize and disentangle the dynamic nature of complex diseases informed by the determination of the extent of genetic and/or environmental disease mechanisms while acknowledging historical elements of nomenclature. Recognizing that multiple factors need to be taken into account when defining and describing diseases, we propose here (Fig. 2) an overview of the breadth of factors contributing to complex diseases.

The Complex Disease Model looks to incorporate the breadth of potential factors driving complex disease etiology. After we modeled the breadth of factors pertinent to Complex Disease etiology, we developed a priority list of factors to be reviewed and then integrated in the Human Disease Ontology. The list of factors was expected to evolve as we continued to now examine disease etiology through this complex model. The first step for investigating each factor was to discuss, and determine how each factor can be captured in a rigorous way during our monthly team meetings.

Complex factors are being integrated in two ways. Firstly, for each reviewed disease several genetic factors are included (molecular mechanism of disease, genes/variants, mode of inheritance, monogenic, digenic or polygenic) in the textual definition and via annotation that specify the genetic factor, e.g. paternally inherited, digenic, autosomal recessive inheritance). Genetic susceptibility factors have already been captured in the project’s ‘omim_susceptibility’ import file, defining the relationship (by ‘contributes to condition’ relationship) between the genetic susceptibility factor, e.g. ‘glioma susceptibility 3’, and a disease, e.g. ‘high grade glioma’. In 2022, we established and are continuing to populate a Disease Driver ontology, that illustrates the various Environmental Drivers. Across the Disease Ontology space, we have already incorporated: Age of Onset and Immune System factors. In order to incorporate ‘age of onset’, we initially mined the Human Disease Ontology textual definitions where age of onset was noted, then incorporated this ‘age factor’ by defining the disease to factor relationship through an annotation on each of the pertinent disease records, utilizing the Human Phenotypes’ Onset categories. One area of focus in this work, included working with the CIViC and ClinGen resources to annotate ‘Pediatric onset’ where applicable, across the Human Disease Ontology. A few years ago, immune system factors were annotated across the Human Disease Ontology in collaboration with the Immune Epitope database. For example, ‘Loeffler syndrome’ (DOID:9503), an ‘eosinophilic pneumonia’, which is considered to also be an allergic reaction, is co-classified as an immune system disease, specifically an ‘allergic disease’ (DOID:1205) by the annotation in the ‘Loeffler syndrome’ (DOID:9503) record by defining the disease to factor relationship as: ‘has symptom’ some ‘allergic reaction’.

Secondly, we expanded our curation efforts on specific epigenetic, environmental and additional genetic factors each quarter of the year. In 2022–2023, we are augmenting the Human Disease Ontology’s classification, reviewing and integrating digenic and polygenic diseases; RNA-associated diseases (miRNA, lncRNA, piRNA). This work involved the integration of new disease terms, the revision of existing terms (synonyms, term nomenclature, annotations to define the disease to factor relationship, e.g. disease ‘has material basis in’ some ‘digenic inheritance’). Subsequently, complex factor integration will focus first on broadening the annotation of environmental drivers of disease and examining the stage of development at exposure.

The intent of this effort is to assess the breadth of possible factors, to then determine how (and if) the factors can be rigorously captured in the disease classification. We recognize that not all of the complex factors identified may be able to be codified in this manner. The complex disease model here reported incorporates the broad range of factors driving human disease, tested against examples with challenging complexity of genetic and environmental factors that should lead to a robust tool that will be able to capture complexities of other factors driving human disease.

**Fig. 3:**
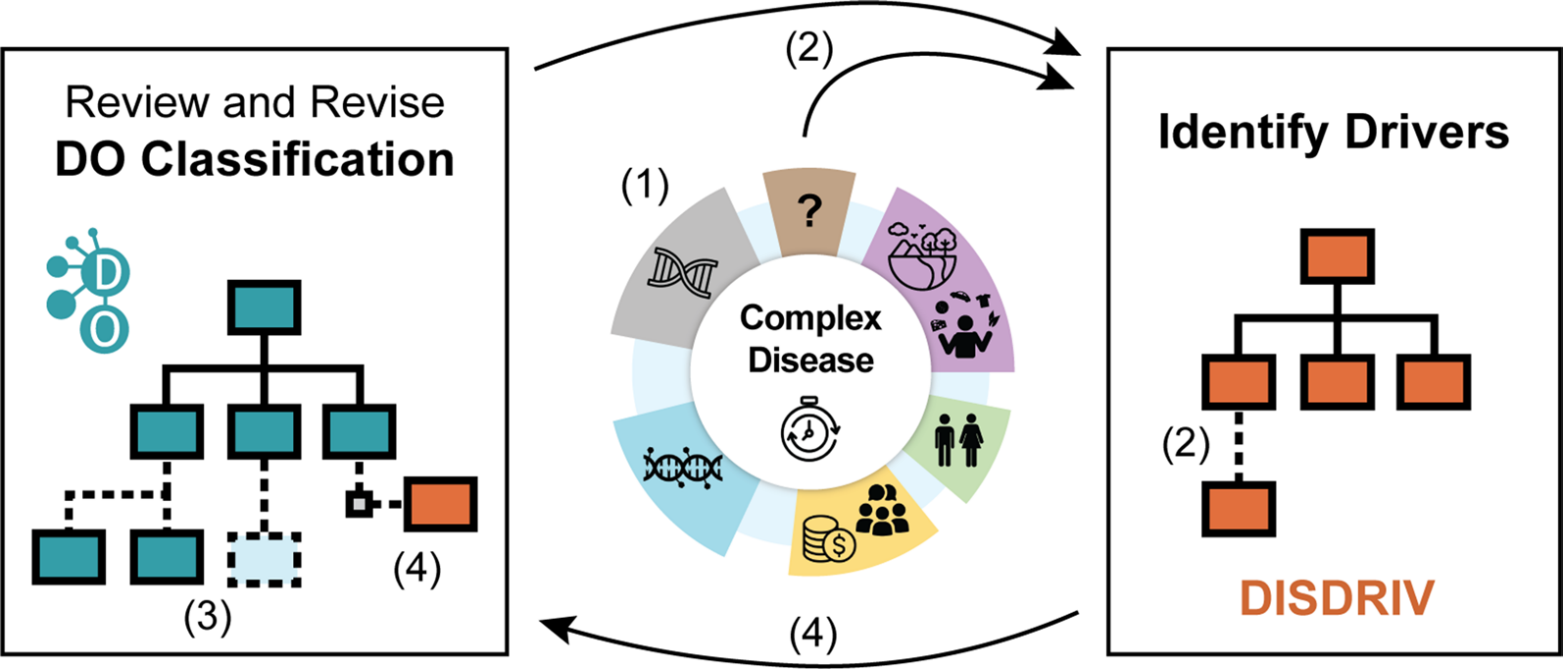
Driver Assessment to DO classification workflow: the established workflow enables testing of the Complex Disease model through the identification of drivers for specific complex diseases. Assessment results in (1) identifying the genetics, epigenetics, social determinants of health, environmental drivers and other host factor drivers for a disease (2) updating the disease driver terms in the DO’s DISDRIV ontology, (3) revision of the DO classification (addition or removal of disease terms, defining, and (4) defining disease to driver relationships by including an ontology axiom statements to define the disease to driver association (e.g. FAS ‘has_disease_driver’ alcohol)

This modeling effort involved systematically identifying the contributing pathogenic effects from a thorough literature review of genetic, epigenetic, host, and environmental factors to the etiology of human diseases and determining whether particular driving factors manifest as clinically recognizable disease subtypes. Ongoing assessment of disease drivers and the subsequent revision of the DO’s disease classification is outlined in the established workflow (Fig. 3), which enables testing of the Complex Disease model through the identification of drivers for specific complex diseases.

Recognizing the dynamic understanding of disease and acknowledging historical elements of nomenclature, it was apparent that the DO’s ‘complex disease’ representation would need to evolve. The next step in this process was to examine the etiology of a number of complex diseases through the lens of this model in order to assess and confirm that this model would enhance the representation of the breadth of genetic, environmental, and pathophysiologic mechanisms of asthma, diabetes mellitus, and fetal alcohol syndrome within the DO’s disease classification system.

### Asthma (DOID:2841) as a defining model

Asthma, historically, has been variously classified in the past. “Asthma” is derived from the Greek meaning *short of breath*, and was further refined by Salter in his work entitled "On Asthma and its Treatment," which focused on the episodic nature of the disease characterized by reversible smooth muscle bronchiole constriction [22]. Osler in 1892 described many of the features that highlight the complex array of familial, environmental, and pathophysiologic features lending to classification problems for more than 100 years. For example, he described spasm and swelling, resembling hay fever, running in families, childhood and older variants, and environmental triggers such as hay, dust, cold and emotional triggers such as fright or emotions [23]. Although much more is now understood about asthma, from a cellular and genetic basis of the host including airway remodeling, abnormal barrier function and innate immune immunity that can explain the variability of response to infection, triggers, and seasonality [24], prior to this modeling the DO’s classifying systems did not encompass the heterogeneity of this disease and it’s causes.

The definition of asthma is based on symptom linked physiology. However, asthma presenting symptoms are not specific for the underlying pathophysiology. Within the scope of our current understanding, the most direct definition would be to refer to asthma as “the disease that includes the physiologic abnormality of airflow limitation, which is variable over short periods of time” [25]. Moreover, it is also important to consider that certain clinical signs of asthma such as wheezing (the manifestation of airflow limitation) can be seen in other conditions such as infections (bronchiolitis) and cardiac causes such as congestive heart failure, as examples which can be mistakenly diagnosed as asthma.

Applying the Complex Disease model to diseases beyond the examples provided here, would involve a review of the breadth of factors associated with a disease, as reported in literature and reported in authoritative biomedical databases and NIH Institute websites. For example, in order to integrate the connection between epigenetic modifications, such as DNA methylation, multiple resources would be identified and reviewed, the list of related diseases would be collated and the associated provenance, to document the data sources, would be curated and ultimately annotated into the Human Disease Ontology. Each feature that is annotated in the Human Disease Ontology, is defined by the usage of concepts defined in their respective ontologies. For example, in order to annotate the connection between an epigenetic modification and a disease, the relationship would be defined by a term from the ‘Sequence types and features ontology’, such as ‘methylated_adenine [SO:0000161]. Applying the developed model to other complex diseases, such as overlap syndromes and chronic obstructive pulmonary disease (COPD), would involve a literature review to determine if any nomenclature changes have been published that are not yet incorporated into the Human Disease Ontology. Determine if endotypes describing pathophysiological, mechanistic pathways have been devised. Through this review a list of associated factors is tallied, discussed and then added to the Human Disease Ontology.

## Results

The clinical conditions (asthma, diabetes and fetal alcohol syndrome) were selected for testing the model as their etiology involved at least two factors, a genetic susceptibility and an environmental driver. Additionally, from the breadth of conditions considered, we selected those where members of our team have extensive knowledge; where sufficient and recently published literature was available and where the complexity of the condition was broad enough to test the model.

Modeling of complex disease evolved within the Human Disease Ontology project, building on the DO Clinician group's work over the past five years, in which we systematically reviewed and revised the DO’s classification system for syndromes, genetic diseases and physical disorders. This work involved the development of a system to enable differential diagnosis of complex genetic diseases, by redefining etiology classifications for non-monogenic diseases, to enable precise etiology definitions. For example, previously in the DO Prader-Willi was defined as a chromosomal disorder and the revised classification captures multiple possible etiologies of a ‘loss of function variant’ in combination with either maternal_uniparental_disomy, paternal_variant, chromosomal_deletion or chromosomal_translocation. Through this work, to improve the DO classification of genetic causes of disease it became clear the DO classification strategy did not have a way to rigorously capture and convey non-genetic (social, environmental, and other) factors pertinent to disease etiology.

The development of the Complex Disease model evolved through months of discussions among the Human Disease Ontology’s Clinical team (co-authors of this manuscript), as we were charged with considering how the Human Disease Ontology could contribute to a more in depth understanding of the complex factors involved. We established a list of common diseases involving varied and complex etiologies involving nuanced etiologies, including lysosomal storage diseases, Parkinson’s disease, fetal alcohol syndrome, autism, amyloidosis, diabetes, and asthma. Based on the group’s interests, areas of expertise and the likelihood of each example to seriously challenge the scope of the model, we selected to explore the three examples of diabetes, asthma and fetal alcohol syndrome. We intentionally picked challenging case-studies to be sure the resulting model would be robust.

### An evolving understanding of asthma

The first step of defining asthma as a complex disease model was to examine current asthma classifications in DO and across clinical vocabularies (ICD, SNOMED CT, OMIM) [26–28], and to research literature to integrate start-of-the-art knowledge on genetic susceptibilities, environmental drivers, severity, endotypes and how researchers and clinicians are defining asthma subtypes (Fig. 2).

**Fig. 4:**
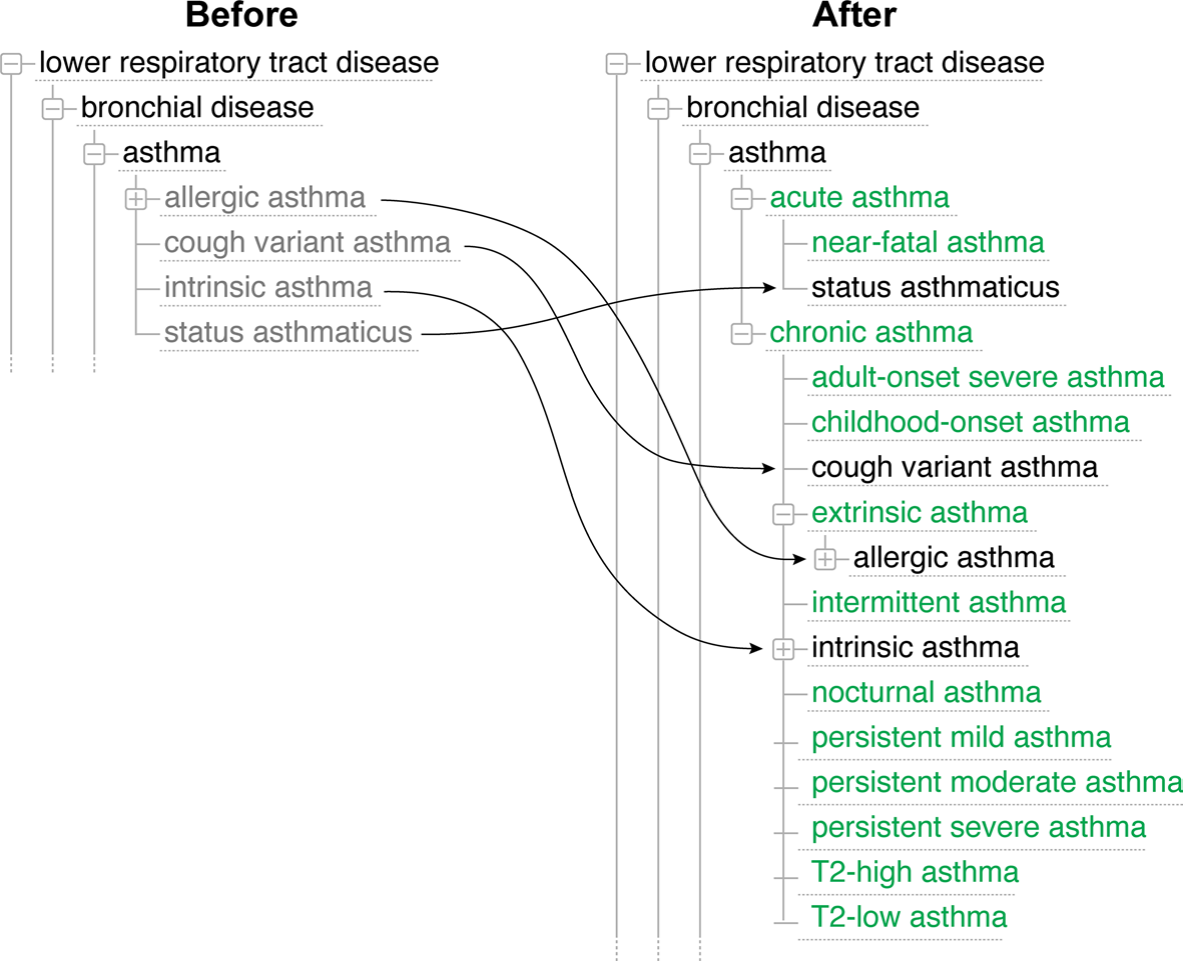
Asthma classification. **a** Asthma classification before refactoring; **b** Refactored asthma classification. Including endotypes, expansion of subtypes

The complexities revealed by this representation in the DO informed the development of a modularized, structured model. We hypothesized this method could be used to model a streamlined, integrative approach for subtyping complex human diseases by defining diseases sharing similar molecular variant types, genetic susceptibilities and/or environmental drivers from authoritative clinical, genetic, and phenotype resources to identify diseases with common underlying etiology. This approach will enable researchers and clinicians to explore common, rare, and complex disease drivers across genetic diseases, syndromes, and cancer and to formulate testable hypotheses to examine mechanisms of pathogenesis (Fig. 4).

Modeling the heterogeneity of asthma has more recently evolved to encompass distinct pathophysiological, mechanistic pathways (endotypes) and variable clinical presentations (phenotypes) thus shifting therapy paradigms leading to precision medicine approaches [29–31]. Asthma has a constellation of phenotypes that can be associated with endotypes to guide clinical management.

### Asthma endotypes

Asthma endotypes may be broadly regarded as type 2 (T2) high or Non T2-low. The phenotypes of T2 (high) endotypes include atopic, late onset, and aspirin exacerbated respiratory illness (AERD) and have defined clinical characteristics, molecular mechanisms, biomarkers and natural history. For example, atopic asthma is seen early, is sensitive to steroids, is molecularly associated with allergic sensitization, is associated with biomarkers including high IgE, is readily identifiable and is associated with preservation of lung function to complete the characteristic phenotype. Contrarily, Non T2 low endotypes include phenotypes such as non-atopic individuals, smokers, individuals with obesity related illnesses and the elderly. For example, for smokers, clinical characteristics would include older adults with a molecular basis of oxidative stress, biomarkers of induced sputum neutrophils, and a clinical course with more frequent exacerbations and lower lung function. This strategy to associate molecular mechanisms to phenotype and asthma endotypes allows us to describe distinct pathophysiologic mechanisms at a cellular and molecular level with implications for treatment and prognosis [30]. Integrating endotypes into current disease etiology modeling will incorporate the multifactorial genetic, environmental and pathophysiological mechanisms of disease causation [32].

### Diabetes

#### History of diabetes nomenclature

Reuse of clinical terms complicates disease nomenclature, as exemplified by the usage of highly similar names such as diabetes insipidus (DI) and diabetes mellitus (DM) to represent distinct disease entities associated with excessive urine output.

Historically, the two conditions were differentiated based on the work of Thomas Willis (1670 s) followed by Johann Peter Frank (1794) [33, 34]. While DM had already been identified as a disease in ancient Egypt, Greece, and Asia, DI was described several thousand years later. Thomas Willis first noted the sweet taste of urine from polyuric subjects compared with healthy subjects, leading to the differentiation of DM from the rare DI. Johann Peter Frank’s description of polyuric patients with not sweet urine led to the terminology of DI. The historical milestones identifying the different forms of DI evolved over time, beginning with the observation by DeLange in 1935 that some patients with DI did not respond to pituitary extract and thus that DI was nephrogenic in origin rather than central [33]. Subsequently, in 1947, Williams and Henry introduced the term “nephrogenic diabetes insipidus” for the congenital syndrome characterized by polyuria and renal concentrating defect but unaffected by vasopressin. Recognizing this important history warrants caution, noting that current medical usage of the word ‘diabetes’ is generally assumed to refer to disorders of glucose regulation. While usage of the word ‘diabetes’ in literature may refer to either DM or DI. Given the nomenclature history of diabetes [35], we additionally reviewed and updated the classification of DI to provide an up-to-date disease classification of both DM and DI.

Previous diabetes mellitus nomenclature revisions have included updates from ‘type I diabetes mellitus’ or ‘insulin-dependent diabetes mellitus’ to ‘type 1 diabetes mellitus’ and from: ‘type 2 diabetes’, non-insulin-dependent diabetes mellitus’, type II diabetes mellitus’ to ‘type 2 diabetes mellitus’. Molecular subtypes as defined by the Online Mendelian Inheritance in Man (OMIM) [27] were added to the DO for type 1 diabetes mellitus (DM1). OMIM type 2 diabetes mellitus (DM2) subtypes, which define susceptibility phenotypes, will be added to the DO when additional evidence of genetic association is defined in OMIM.

**Fig. 5:**
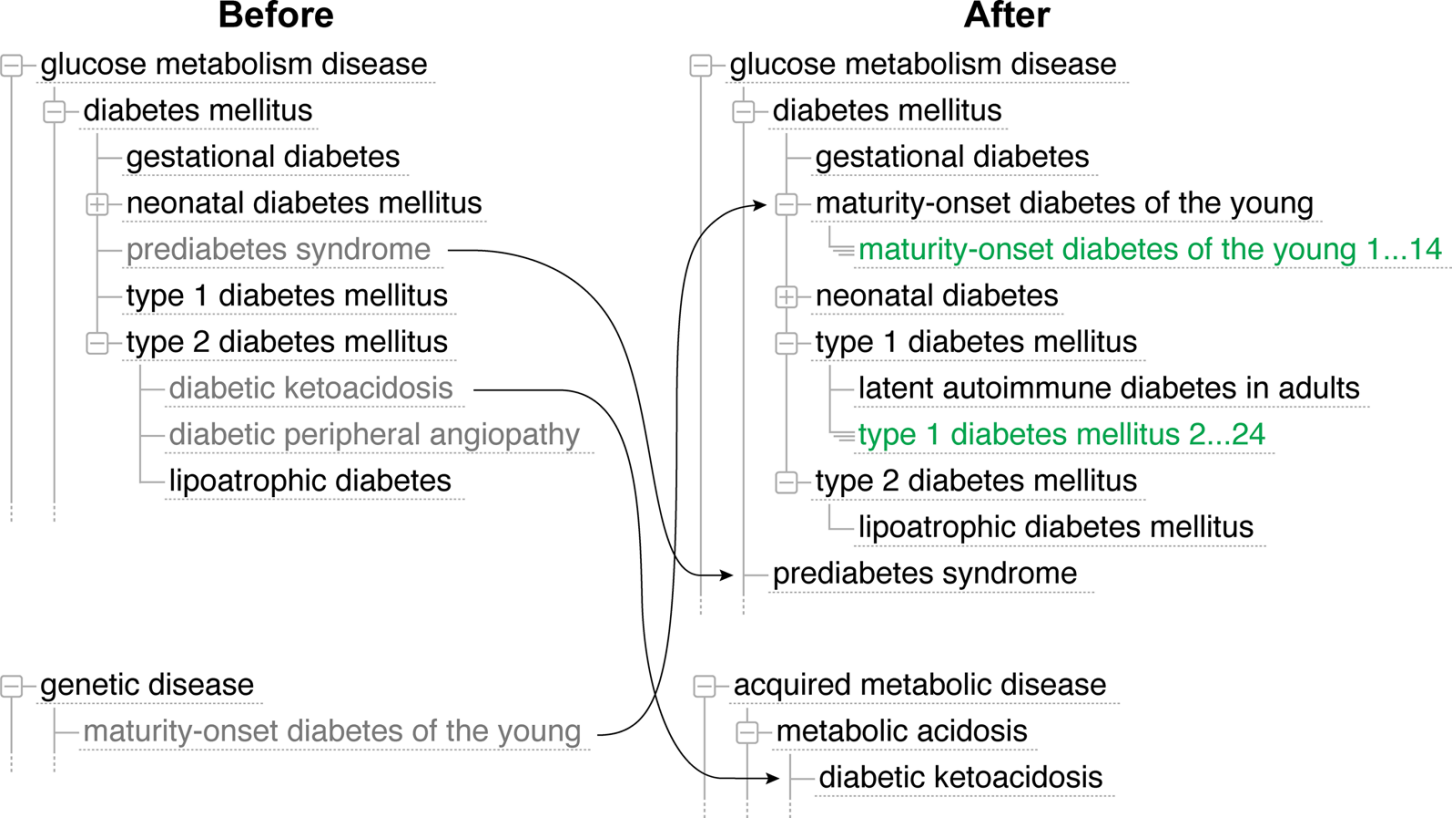
Diabetes mellitus reclassification. Showing the reclassification of diabetes mellitus following the recent review

Recent re-evaluation of diabetes as a complex disease resulted in a DM2 reclassification (Fig. 5). The review identified 14 molecular subtypes of MODY (maturity-onset diabetes of the young, DOID:0050524) and the reclassification of ‘latent autoimmune diabetes in adults’ (DOID:0080846) as a subtype of ‘type 1 diabetes mellitus’. Review of DI (DOID:9409), a subtype of ‘kidney disease’ (DOID:557), identified four subtypes: central diabetes insipidus, nephrogenic diabetes insipidus, gestational diabetes insipidus, and dipsogenic diabetes insipidus.

### Fetal alcohol syndrome as a test for the complex disease model

History of Disease. Fetal alcohol spectrum disorder (FASD (DOID:0050696)) is the name given to a constellation of signs and symptoms associated with prenatal ethanol exposure. Fetal Alcohol Syndrome (FAS) (DOID:0050665) is the most severe manifestation of FASD.

FAS was first described in 1968 in 127 children born to alcoholics in France [36]. It was more widely recognized following an article published in the Lancet in 1973 that described common features of 8 children born to alcoholics of three different ethnicities [37].

These children were born to women who were chronic alcoholics throughout pregnancy, so they were constantly exposed in utero. The manifestations of FAS in each case were similar, leading to the establishment of criteria needed to make the diagnosis. The criteria are: (1) documentation of growth deficits (weight, length, head circumference), (2) documentation of the following three facial features: smooth philtrum, thin upper lip, short palpebral fissures, and (3) documentation of central nervous system abnormalities which can be structural, neurological or functional) [38]. Maternal alcohol use can be either confirmed or unknown. Because the three criteria may be fulfilled at different stages of development, FAS is most frequently diagnosed at school age when behavioral problems are noted by a teacher. A FAS diagnosis can be accompanied by a variety of other structural and function deficits in other organs, including the cardiovascular system, genitourinary system, sensory systems (most notably auditory) and the autonomic nervous system.

The term, “Fetal Alcohol Syndrome (FAS)” was coined in a 1973 article in the Lancet by Smith and Jones to describe common physical features of children born to alcoholic mothers. The first recognition of a pattern of specific deficits seen in children of women who consumed alcohol heavily in pregnancy was made by Lemoine et al. in 1968. However, as this observation was published in French and did not give a name to the pattern, it was not recognized until the 1973 publication in Lancet. Initially, it was thought to be due to malnutrition but is now recognized as an effect of ethanol exposure. Because the maternal drinking in these initial cases was continuous and ongoing during pregnancy, new questions of dose and timing of exposure arose. New research then began to fully describe FAS, determine its mechanism (still unknown), and to determine the effects of dose and timing of dose to the outcome. Over the course of many years of research, a plethora of terms was introduced to describe the range of outcomes from a range of ethanol exposure patterns during pregnancy. These terms initially included fetal alcohol effects and alcohol related birth defects. In 1996, the Institute of Medicine developed a diagnostic nomenclature that included the terms of FAS, partial FAS, alcohol-related birth defects (ARBD) and alcohol-related neurodevelopmental disorder (ARND). The category, “Fetal alcohol effects” subsequently was phased out.

The diagnostic criteria for FAS according to the Institute of Medicine (now the National Academy of Medicine) is the presence of characteristic facial features (short palpebral fissures, a flat elongated philtrum, and a thin upper vermillion lip border), growth impairment (< 10th percentile weight) and central nervous system deficits (head circumference < 10th%, poor suck, weak cry, mental retardation). The diagnostic term of partial FAS is applied when facial features are present along with either growth deficits or physical central nervous system (CNS) deficits (e.g. microcephaly) or characteristic neurobehavioral problems are present. ARBD is used when congenital structural defects (cardiac, kidney, auditory) are present along with a history of maternal alcohol consumption modified in 2005 to include the requirement that facial features of FAS be present. The diagnosis of ARND requires evidence of either physical CNS deficits or neurobehavioral deficits similar to those in FAS are present along with a history of maternal alcohol use in pregnancy.

The term, now in wide usage, of Fetal Alcohol Spectrum Disorder (FASD), includes FAS, partial FAS, ARBD, ARND, and other outcomes thought to be related to alcohol use during pregnancy. There are no established definitive diagnostic criteria for diagnosing FASD. Interestingly, there are several ICD-10 codes for ethanol use/exposure during pregnancy: P04.3 Newborn affected by maternal use of alcohol; Q86.0 Fetal alcohol syndrome (dysmorphic); 099.31 alcohol use complicating pregnancy, childbirth, and the puerperium, alcohol use complicating pregnancy, unspecified trimester; 035.4XX0 maternal care for damage to fetus from alcohol, not applicable or unspecified. FASD is not an ICD-10 code**.** The lack of inclusion in coding systems impedes diagnosis and treatment.

Ethanol, the environmental driver. It became apparent that the diagnosis of FAS was not capturing all infants affected by maternal ethanol exposure. Manifestations could vary depending on the pattern of drinking (a woman who drinks 5 drinks per week may drink all 5 on one day (binge episode) or 1 drink per day for 5 days of a week. Both result in a 5 drinks/week dose, however, binge drinking is more harmful to the fetus. Various disease names were applied depending on the predominant effects: alcohol related birth defects (DOID:0050668), alcohol related neurodevelopmental disorder (DOID:0050667), partial fetal alcohol syndrome (DOID:0050666), neurobehavioral disorder with prenatal alcohol exposure (DOID:00810520). The diagnosis of FASD has been complicated, but recent progress has been made in defining how these diagnoses are to be made. Hoyme et al. published a diagnostic criteria for each of the syndromes for the clinical diagnosis of FAS and FASDs [39]. They have all now been placed under the term Fetal Alcohol Spectrum Disorder. The DO was augmented following this review, by the addition of neurobehavioral disorder with prenatal alcohol exposure (DOID:00810520), the addition of age of onset annotations and the annotation of alcohol as an environmental driver. Age of onset was added utilizing the Relations Ontology term: 'existence starts during' and onset as defined in the Human Phenotype Ontology, 'Pediatric onset', where the onset of disease manifestations occurs before adulthood, defined as before the age of 15 years (HP:0410280). The causal relationship between alcohol and FASD was defined with the addition of a new Relation Ontology term, ‘has disease driver’ (RO:0007001) and term ‘alcohol’. In addition to the factors known to influence development of FASD, there is growing evidence of other environmental, host, ‘social’ determinants of health, genetic and epigenetic contributing factors that need further studies to elucidate these relationships [39].

Other environmental drivers. In some studies, maternal smoking is always associated with FAS. Recent data suggests that dietary factors may also influence the impact of alcohol: both polyunsaturated fatty acids and choline have been shown to both prevent the effects of alcohol on the developing fetus, as well as to repair some of the damage. In a study from the Ukraine, a randomized controlled trial of choline supplementation to children who had the diagnosis of FAS/FASD resulted in a modest improvement in outcome [40, 41].

Other Host Factors. The stage of development has different effects on the fetus (first trimester only, third trimester only, all three trimesters, or only before recognition of pregnancy). Exposure during the first trimester is required for the facial dysmorphology that is required for a diagnosis of FAS. However, the brain remains vulnerable throughout pregnancy. In a sheep model, effects of second trimester only and third trimester only binge exposure has been described to lead to different effects on outcome [42].

“Social” Determinants of Health. Drinking and heavy drinking varies by ethnicity with rates higher in whites and Native Americans than in Asian Americans and Hispanics [43]. Socioeconomic status also influences drinking behavior amongst women and the health impacts of drinking in a complex way [44]. For the outcome of FASD, several studies based on single cities demonstrate the complexity of the interaction between social determinants of health and FAS/FASD. Late recognition of pregnancy and higher dose of consumed alcohol appear to be two common risk factors [45–48].

Genetics. Genetic influences must consider both the genetic makeup of the parents as well as those of the fetus. Genes influencing the development of FAS/FASD have mostly focused on maternal alcohol dehydrogenase and acetaldehyde dehydrogenase [49]. Other implicated pathways in the fetus include the retinoic acid pathway, sonic hedgehog and cholesterol homeostasis, nitric oxide synthase I, and platelet derived growth factor/mTOR pathways [49]. Recently, a twin study showed convincing evidence that genetics plays a part in FAS/FASD. The study showed decreasing concordance with decreasing genetic relatedness [50].

Epigenetics. Multiple genes in many pathways have been found to be epigenetically regulated by fetal ethanol exposure [51, 52]. Examples of the impact of alcohol induced alteration of gene expression are on the cortical thinning present in FAS/FASD [53] and hypothalamic-pituitary axis [54].

In summary, the model of complex disease for FAS/FASD makes several points clear, that there are multiple opportunities to intervene that may affect the impact of ethanol exposure to reduce the burden of FAS/FASD. However, the simplest solution might be to prevent prenatal alcohol exposure in the first place.

## Discussion

The devised complex disease model provided a framework for appropriately reclassifying diseases to reflect modern understanding of complex diseases. The testing of the complex disease model with the asthma, diabetes and fetal alcohol syndrome use cases suggested that the model does include all the necessary elements and also revealed additional challenges. This complex disease model is a framework that supports the future integration into the Human Disease Ontology of the expanding knowledge about causes of disease. Use of the model to support classification in the Human Disease Ontology, enhances the ability of the DO to show and examine multiple types of connections between diseases based on shared drivers and mechanisms. This enhances the utility of the DO as a resource for differential diagnosis.

### Disease within a disease and potential diagnostic errors

The wide range of definitions of asthma, and the finding of the symptoms of asthma in other conditions demonstrated another challenge to the model: the recognition that one condition could be the result of, or the symptom of, another condition. The concept of “disease within a disease”, is an ongoing challenge for any disease ontology. For example, VACTERL, the association of vertebral defects (V), anal atresia (A), tracheoesophageal fistula with esophageal atresia (TE), radial or renal dysplasia (R), cardiac malformations (C) and limb anomalies (L), is a often a feature in Fanconi anemia diagnosis [55]. Likewise, bilateral generalized polymicrogyria is also a feature of several genetic syndromes characterized by intellectual disability and multiple birth defects including 22q11.2 deletion syndrome, Adams-Oliver syndrome, Aicardi syndrome, Joubert syndrome, and Zellweger spectrum disorder [56–60]. At its simplest, this is a concept implicitly understood. For example “cough” is a symptom, and it is listed as a diagnosis in our diagnostic coding system in the USA, reflecting the fact that the “diagnosis” of cough leads to certain differential diagnosis and options for therapy. Cough can have behavioral, neurological, mechanical, allergies or infectious causes. At a more complex level, “asthma” may be the diagnosis of an individual who proves to have alpha 1-antitrypsin deficiency or Cystic Fibrosis. In some cases the “diagnosis” of asthma may prove to be incorrect, but if the patient meets criteria for the diagnosis of asthma and responds to therapy for asthma, it may be more correct to recognize asthma in that case as a manifestation of the underlying disorder. Thus, it is imperative to develop diagnostic guidelines integrating this complexity in order to advance diagnostic capabilities. The issue at hand is that clinical diagnosis is susceptible to implicit bias, as a matter of perspective. That is, fitting observations into conclusions that are familiar or that have previously been seen. However, the imperative is that diagnosis must extend beyond first examination with a patient in order to discover the root cause of the primary etiology. Otherwise, the diagnosis is not complete. One must consider that the presentation may be atypical and may be the outcome of another disease.

Consequently, complex disease classification must transition to recognize and differentiate the distinctness of overlap syndromes, by integrating the genetic complexity (e.g. gene interaction information, non-synonymous variants impacting protein structure and function, epigenetics) and capturing the disease heterogeneity when a disease shares features of other distinct diseases, resulting in a unique clinical phenotype and outcome [61]. For example, the classification for chronic obstructive pulmonary disease (COPD) should expand to include COPD subtypes, including asthma-COPD overlap syndrome, bronchiectasis-COPD, fibrosis-COPD and OSA-COPD. On the other end of the classification spectrum, a patient with clinical features of a genetic disorder deserves a diagnosis even if the causative variant(s) cannot be identified using current methodology, for example, > 6 CALs (cafe-au-lait spots), axillary freckling and dermal neurofibromas should be diagnosed as Neurofibromatosis Type 1 even if there is no identifiable mutation in the NF1 gene [62, 63]. Consider further, environmental exposures may present like a genetic disease, thus without a confirmatory genetic test, there is the risk that a patient will be given an incorrect diagnosis as the true cause has not been revealed. Symptoms that identify diseases, such as wheezing or bronchospasms, complicate diagnosis. For example young children with symptoms of wheezing which when responsive to bronchodilators is called asthma; however, there is also gastroesophageal reflux, cystic fibrosis, bronchitis, COPD, food allergy, or cancer to consider.

### Historical definitions and labeling

Further complicating defining complex disease are historical definitions and labeling which are commonly used by clinicians but may not be easily classified in the Disease Ontology framework as a disease. As an example both bronchospasm and reactive airway disease reflect complex diseases but are not able to be classified in the DO. Bronchospasm occurs when the airways (bronchial tubes) go into spasm and contract. This makes it hard to breathe and causes wheezing (a high-pitched whistling sound). Bronchospasm can also cause frequent coughing without wheezing. Bronchospasm is due to irritation, inflammation, or allergic reaction of the airways [64]. However, bronchospasm is classified as a symptom, not a disease. Often, the term "reactive airway disease" is used when asthma is suspected, but not yet confirmed. Reactive airway disease in children is a general term that does not indicate a specific diagnosis. It might be used to describe a history of coughing, wheezing or shortness of breath triggered by infection [65]. Ideally, the future of complex disease classification would be driven by an approach where a string of entities including mechanism/variant subset, gene, phenotype, and disease are linked together to enable examination of patterns for diagnostic assessment. An initial step in this direction is to capture these disease “features” within the Human Disease Ontology, by defining ‘disease has feature’ relationships to identify when one disease has a feature of another disease. Applying this integrative model to other complex diseases [66], such as diabetes, would involve capturing the pool of mediating/modifying genes along with the mechanisms of gene-environment interaction in order to explore phenotypic and outcome differences and gain a greater understanding of related exposure attributes.

The Disease Ontology reflects the changing oncological status of diseases, when studies report a change in the WHO grading of a neoplasm for example, when new understanding of a disease’s pathophysiology is reported, or in order to capture when two diseases are closely associated. Utilizing descriptive relationship terms, such as ‘disease has basis in’ further enables the capture of connections between two diseases.

This work may involve the reclassification of a disease (from benign to cancerous) or the inclusion of both benign and a cancerous disease terms. Integrating updates from authoritative classifications, such as the 5th edition (2021) of WHO classification of CNS tumors [67], involves careful review of the current disease classification. For example, in cases where our understanding of a disease has been revised, where a previously low-grade tumor, has been reassessed from a WHO grade 0 to a WHO grade 3. Regarding the disease classification, if a particular neoplasm can be either benign or malignant, then we would create two distinct disease terms within the ontology, to represent each of the WHO grades. If the WHO grade of a neoplasm entirely switched from a benign disease to a malignant disease, that specific disease term would be reclassified to the ‘cancer’ branch of the Human Disease Ontology.

## Conclusions

Any model of complex disease must be flexible to represent both our current understanding of pathophysiology as the interaction of the effects of genes and environmental challenges including other organisms and toxins. The model must be general enough to include disorders that are primarily genetic and those that are primarily driven by environmental factors, and yet have sufficient specificity that will allow for the understanding of each condition to be comprehensive and detailed, in order to support further research into etiologies and the development of therapies. The model needs to recognize old and even outmoded or disproven ways of defining conditions, in order to maintain access to older literature using those definitions and to maintain access to important knowledge against which we might test new hypotheses.

Our testing of the asthma use case illustrated the importance of flexibility in the model for representing both the “condition” (asthma) and an “attack” or episode of the condition. The testing highlighted the challenges of the use of the words “driver” and “trigger” in literature, with “trigger” typically used in context of an event or episode and driver used both in the sense of ‘contribution to the underlying condition’ and to ‘manifestation of symptoms’. The iterative process of model development and use case testing highlighted the challenge of differentiating between drivers and triggers of disease. We devised ‘disease driver’ to be defined as the non-reversible entity that is causally responsible for the occurrence of the disease and a ‘disease trigger’ as the reversible entity that catalyzes an acute occurrence of a disease [68–70]. We further defined ‘disease driver’ as an environmental or genetic mechanism that directly contributes to the disease state, and ‘trigger’ defined as the mechanism that exacerbates the occurrence of a condition. Disease drivers differ from triggers in that the disease state can be reversed by the removal of a trigger, whereas the disease state will perpetuate whether or not the disease driver is present in the future. This is in contrast to a trigger, for example in allergic asthma, the asthma attack is 'triggered' by specific allergens and the ‘asthmatic attack’ will not persist once the allergen is removed.

## Data Availability

The datasets generated and/or analyzed during the current study are available in the the Disease Ontology GitHub repository, [https://github.com/DiseaseOntology/HumanDiseaseOntology].

## Abbreviations

AERD: Aspirin exacerbated respiratory illness
AMED: Aplastic anemia, mental retardation, and dwarfism
ARBD: Alcohol-related birth defects
ARND: Alcohol-related neurodevelopmental disorder
CALs: Cafe-au-lait spots
CNS: Central nervous system
CNV: Copy number variant
COPD: Chronic obstructive pulmonary disease
DI: Diabetes insipidus
DM: Diabetes mellitus
DM1: Type 1 diabetes mellitus
DM2: Type 2 diabetes mellitus
DO: Disease Ontology
DSM: Diagnostic and Statistical Manual of Mental Disorders
FAS: Fetal alcohol syndrome
FASD: Fetal alcohol spectrum disorder
ICD: International Classification of Diseases
ICSI: Intracytoplasmic sperm injection
lncRNA: Long non-coding RNA
miRNA: MicroRNA
MODY: Maturity-onset diabetes of the young
OMIM: Online Mendelian Inheritance in Man
piRNA: Piwi-interacting RNA
SNP: Single nucleotide polymorphism
T2: Type 2
UV: Ultraviolet
VACTERL: Vertebral defects, anal atresia, cardiac defects, tracheo-esophageal fistula, renal anomalies, and limb abnormalities
WHO: World Health Organization
